# One-Pot Synthesis of Polypyrazoles by Click Reactions

**DOI:** 10.1038/s41598-017-12727-3

**Published:** 2017-10-05

**Authors:** Shasha Wang, Bin Cheng

**Affiliations:** Key Laboratory of Beijing City on Preparation and Processing of Novel Polymer Materials, Beijing University of Chemical Technology, Beijing, 100029 China

## Abstract

We developed an efficient one-pot metal-free click polymerization procedure for the synthesis of 3,5-disubstituted polypyrazoles with high yields, high molecular weights, and narrow molecular weight distribution. The method involved two click reactions in a one-pot synthesis. The first reaction was the carbonyl chemistry of “non-aldol” type (condensation reaction of aldehydes with p-toluenesulfonylhydrazide), and the second was a click polymerization reaction of diazo compounds with alkynes. The reactions occurred sequentially by adding the reactants step by step. The diazo compound needed for the second click reaction was generated *in situ* by the first click reaction. The structures of the polypyrazoles were characterized by IR and ^1^H NMR analyses. And their thermal properties and solubility were also tested.

## Introduction

The development of new polymerization reactions for the preparation of heterocyclic polymers is an important area in polymer research. Polypyrazoles, one class of the heterocyclic polymers, have been studied extensively during the past decades due to their excellent film forming properties and high thermal stability^1,2^. Among the synthetic methods available for the preparation of polypyrazoles, a commonly used approach is cycloaddition. Reactions of bis-nitrilimines^3^, bis-sydnones^4^ and bis-azides^5^ with diacetylenes have been used successfully to produce the polypyrazoles. In recent year, it was also successfully produced via click reaction of diazos with diacetyelenes^6^. In addition, Bass and coworkers have used the reactions of bis-hydrazines with acetylenic ketones and esters to prepare thermally stable polypyrazoles^7^. Moore and Mehta have developed a new route to produce polypyrazoles by reacting bis-chlorovinylidene cyanides with diamines^8^. They also improved the thermostability of polypyrazoles by employing a vinylic nucleophilic substitution reaction^2^. However, these methods require the preparation of pure monomers for cycloaddition, which need complex and time-consuming processes and lead to lower overall yields. In addition, the preparation and handling of azides and diazo compounds, which are known to be toxic and potentially explosive, are hazardous. Therefore, there is a need to develop new synthetic method for the preparation of polypyrazoles.

**Figure 1:**
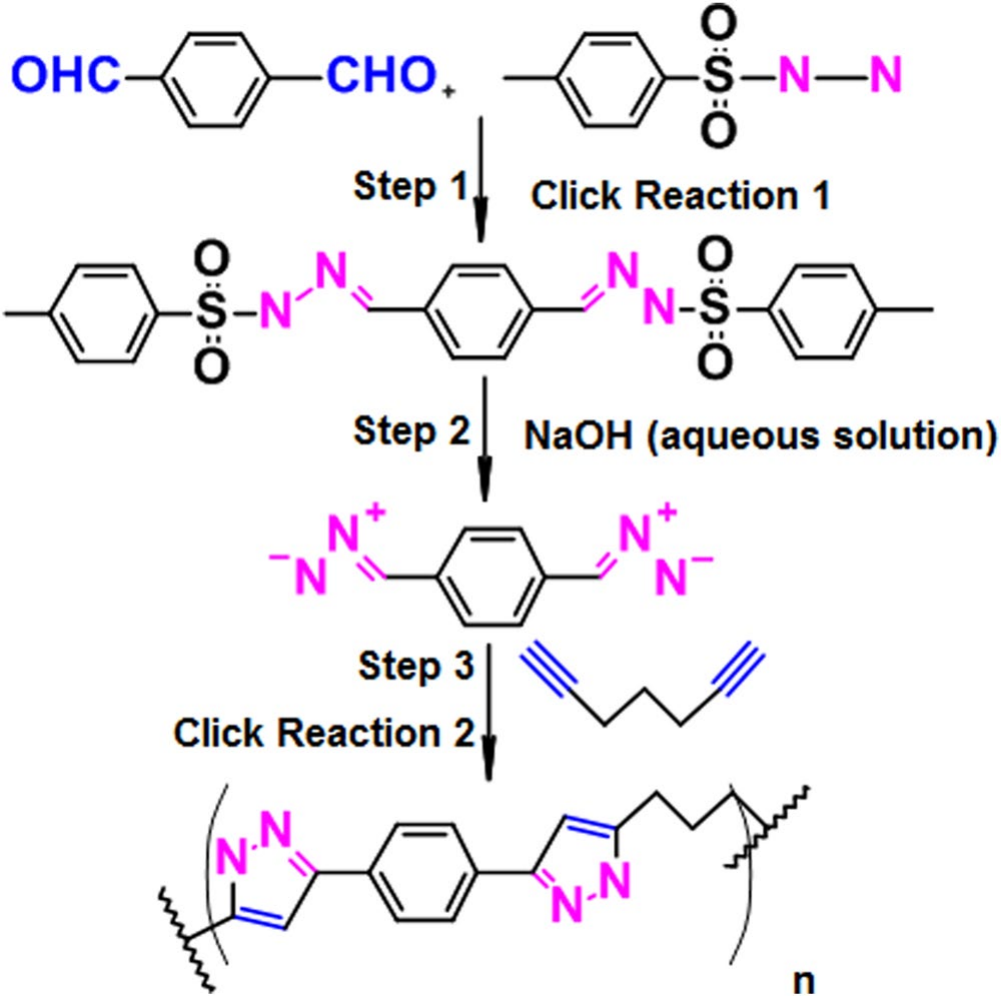
One-pot synthesis of polypyrazoles.

“Click reactions”, first introduced by Sharpless *et al*. in 2001^9^, are chemical transformations with remarkable advantages, including high efficiency, regioselectivity, and atom economy, fast reaction rates, mild reaction conditions, and simple product isolation. They have quickly found widespread applications in various research areas^10–12^. Aggarwal *et al*. succeeded in using the condensation reaction of aldehydes with p-toluenesulfonyl hydrazine and 1,3-dipolar cycloaddition of diazo compounds with alkyne or N-vinylimidazole to prepare pyrazoles in a one-pot process^13–15^. The diazo compounds were generated *in situ* from tosylhydrazone salts and not isolated, which avoided potential toxicity and explosion. In recent years, heterocyclic polymers have been synthesized by click reaction. Particularly, the copper (I)-catalyzed 1,3-dipolar cycloaddition of diazides with dialkynes has been used to prepare poly(triazole)s (PTAs)^16–19^. Moreover, polyisoxazoles were successfully synthesized by click reaction in one-pot manner in our laboratory^20^, which benefited from the high efficiency and regioselectivity of click chemistry. Polypyrazoles are also important heterocyclic polymers and they can be synthesized by click reactions^6^. Inspired by these advances, we investigated the regioselective synthesis of 3,5-disubstituted polypyrazoles by a one-pot three-step process. A dialdehyde was first converted to the corresponding hydrazone via a condensation reaction with p-toluenesulfonylhydrazide. Without isolation, the hydrazone was then transformed to the diazo compound in the presence of aqueous sodium hydroxide. Finally, the *in situ* generated diazo compounds underwent uncatalyzed 1,3-dipolar cycloaddtion with diynes to produce polypyrazoles (Fig. 1). Reaction of diazo compounds with alkynes was chosen to prepare polypyrazoles because alkynes and diazo compounds can be readily activated to generate high reactive species for the cycloaddition reaction^21^. Two click reactions (carbonyl chemistry of the “non-aldol” type and a 1,3-dipolar cycloaddition reaction)^9^ were involved in the one-pot procedure, and no isolation and purification of the *in situ* generated diazo compounds is needed, which avoids the very cumbersome and lengthy process and the toxicity and potential explosion of diazo compounds.

## Results

**Figure 2:**
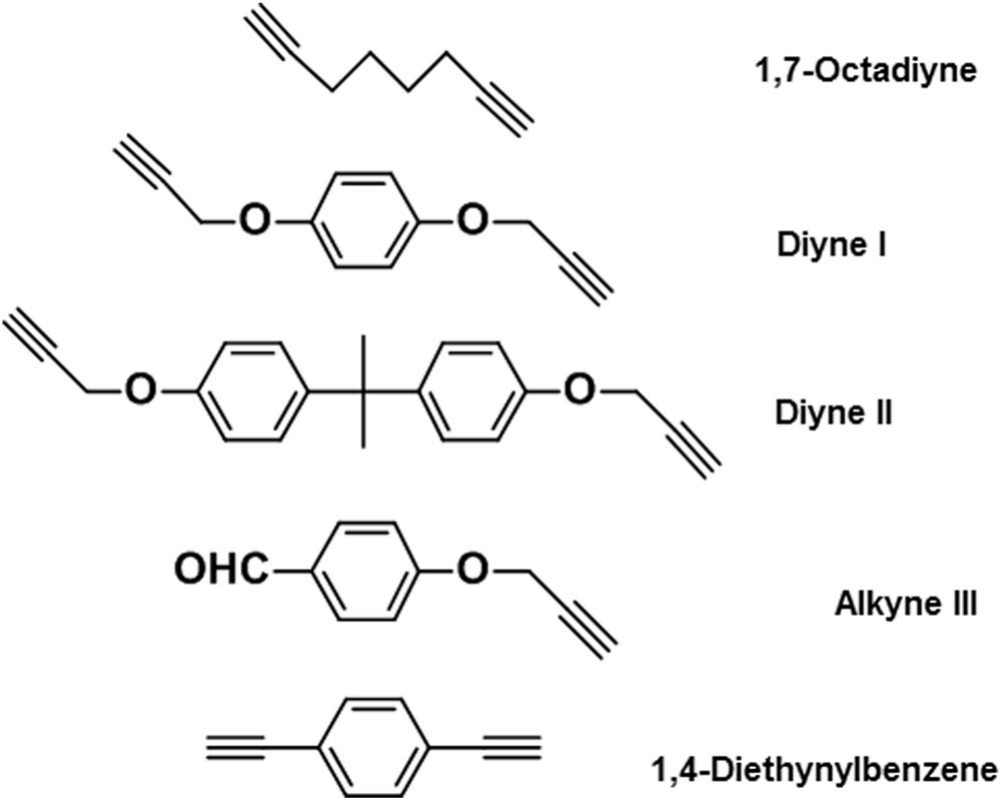
Five kinds of alkyne monomers.

Figure 1 shows the one-pot three steps procedure for the preparation of polypyrazoles. Five different types of alkynes (Fig. 2) were employed to demonstrate the scope of the one-pot strategy, and their polymers are summarized in Table 1. All the polypyrazoles were obtained in high yields, with high molecular weights and narrow molecular weight distribution (Table 2). There is no need to isolate the diazo compounds, which made the reaction process safer. Quantitative conversion of terephthalaldehyde to the corresponding hydrazone and diazo compound was achieved in our procedure, which enabled the addition of stoichiometric amounts of diynes for polymerization.

**Table 1:** Synthesis of polypyrazoles via Click Polymerization^a^.

Entry	Aldehyde Monomer	Alkyne Monomer	Products
1			
2			
3			
4	—		
5			

^a^Polymerization conditions:[Diyne]:[Dialdehyde] = 1:1, 48 h, in 25 ml DMF.

**Table 2:** Yield and Molecular Weight of the Polypyrazoles.

Polymer	*T* ^a^ (°C)	Yield(%)	*M*_*n*_^b^(kDa)	*M*_*w*_^b^(kDa)	*M*_*w*_/*M*_*n*_^b^
**PI**	50	61.02	14.1	16.7	1.2
	60	68.65	12.1	14.7	1.2
	70	59.12	9.0	11.3	1.3
**PII**	50	77.47	11.6	13.9	1.2
	60	84.78	10.9	13.1	1.2
	70	95.01	11.0	13.2	1.2
**PIII**	50	98.92	11.8	14.3	1.2
	60	96.77	11.9	14.4	1.2
	70	98.92	10.9	13.4	1.2
**PIV**	50	76.02	9.3	11.4	1.2
	60	73.10	9.2	11.3	1.2
	70	87.71	8.4	10.3	1.2
**PV**	50	79.23	10.4	13.0	1.3
	60	84.51	10.4	13.1	1.3
	70	82.75	10.2	12.9	1.3

^a^Polymerization temperature. ^b^ *M*_*n*_, number-average molecular weight; *M*_*w*_, weight-average molecular weight; *M*_*w*_ */M*_*n*_, molecular weight distributions were estimated by gel permeation chromatography (DMF, on the basis of polystyrene standards).

**Figure 3:**
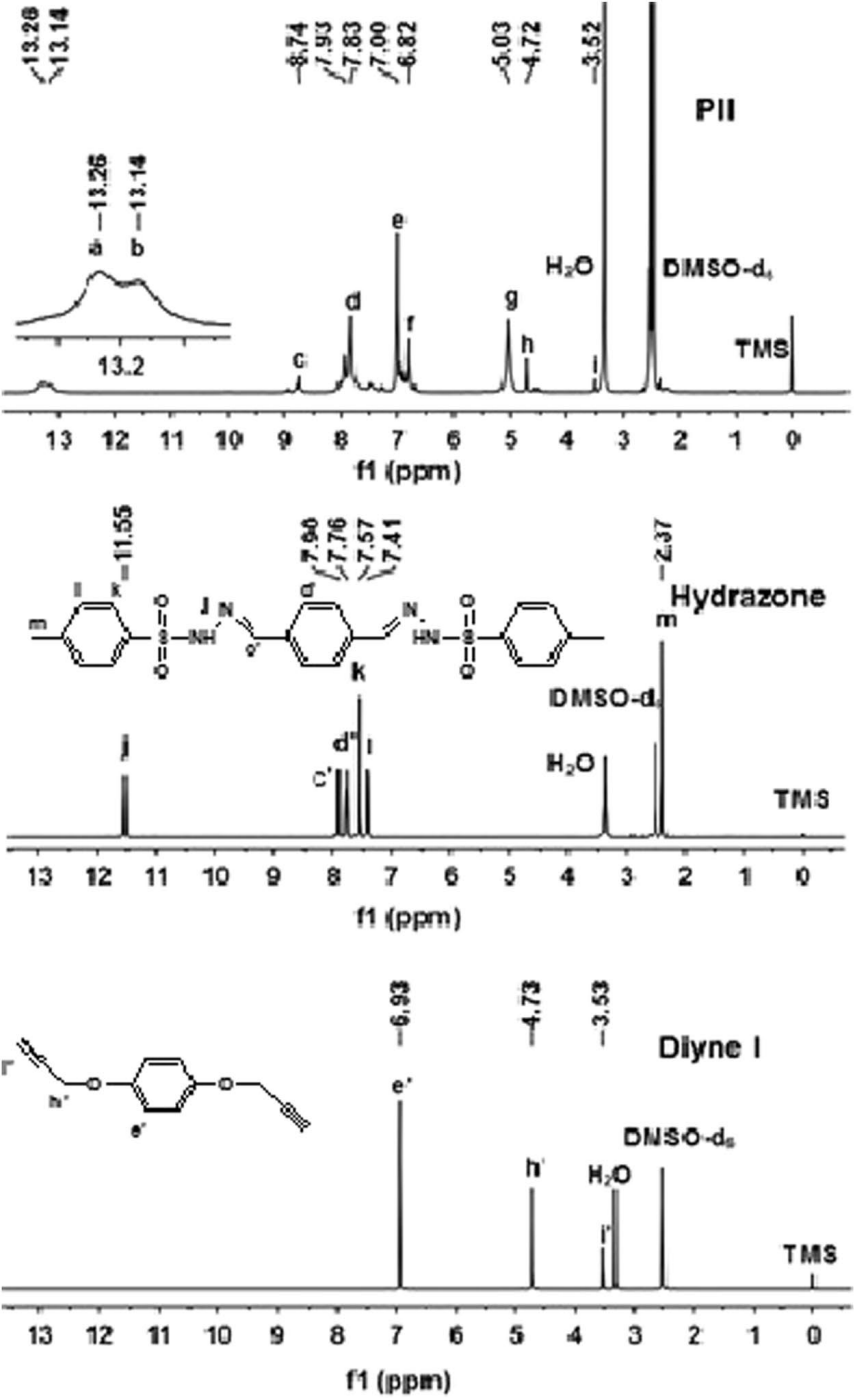
^1^H NMR spectra of polymer **PII**, hydrazone (1,4-tosylhydrazone) and monomer diyne **I** in DMSO-d_6_. The hydrazone (diazo compound precursor) was obtained at the end of the first click reaction of the one-pot procedure.

The structures of the polypyrazoles were confirmed by IR and ^1^H NMR analyses. Figure 3 shows the ^1^H NMR spectra of **PII**, diazo compound precursor (1,4-tosylhydrazone, obtained in an independent experiment) and diyne **I**. The new resonance peaks of the heteroaromatic protons at 6.82 ppm (**f**), protons on nitrogens at 13.26 ppm (**a**) and 13.14 ppm (**b**) were attributed to the pyrazole tautomeric structure, and benzylic protons at 5.03 ppm (**g**) were observed in the spectrum of **PII**. The downfield shift of the benzylic proton signal **h** and significant reduction of the ethynyl proton signal **i** of **PII** along with the disappearance of the N–H signal **j** of hydrazone clearly suggested the formation of **PII**. Signal **d** and signal **e** of the phenyl protons resonances of **PII** were superimposed. Signal **c** was the proton of the terminal phenyldiazomethane, which was from signal **c’** of diazo compound precursor hydrazone (1,4-tosylhydrazone). Only one type of heteroaromatic proton signal was observed, indicating that only 3,5-disubsituted polypyrazole was produced.

**Figure 4:**
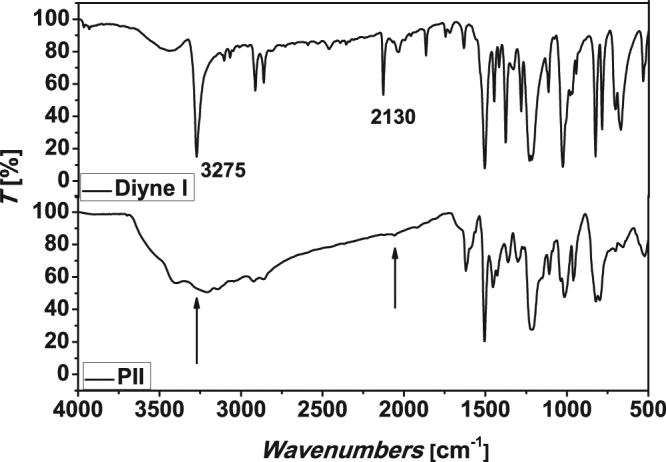
IR spectra of diyne I and polypyrazole **PII**.

Examples of IR spectra of diyne **I** and its polymer **PII** are given in Fig. 4. The spectrum of diyne **I** showed strong absorption bands at 3275 cm^−1^ (≡C-H) and 2130 cm^−1^ (C≡C), which are attributed to the terminal alkynyl group. However, these peaks were very weak in the spectra of polymer **PII**. These results indicated that most of the acetylene groups of the monomers have undergone click reaction to form the pyrazole rings in the polymers.

**Figure 5:**
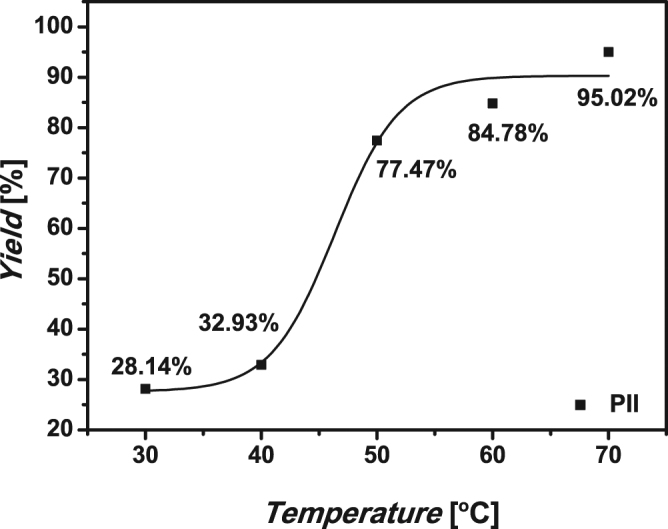
Yields of polymer **PII** at different polymerization temperatures.

**Figure 6:**
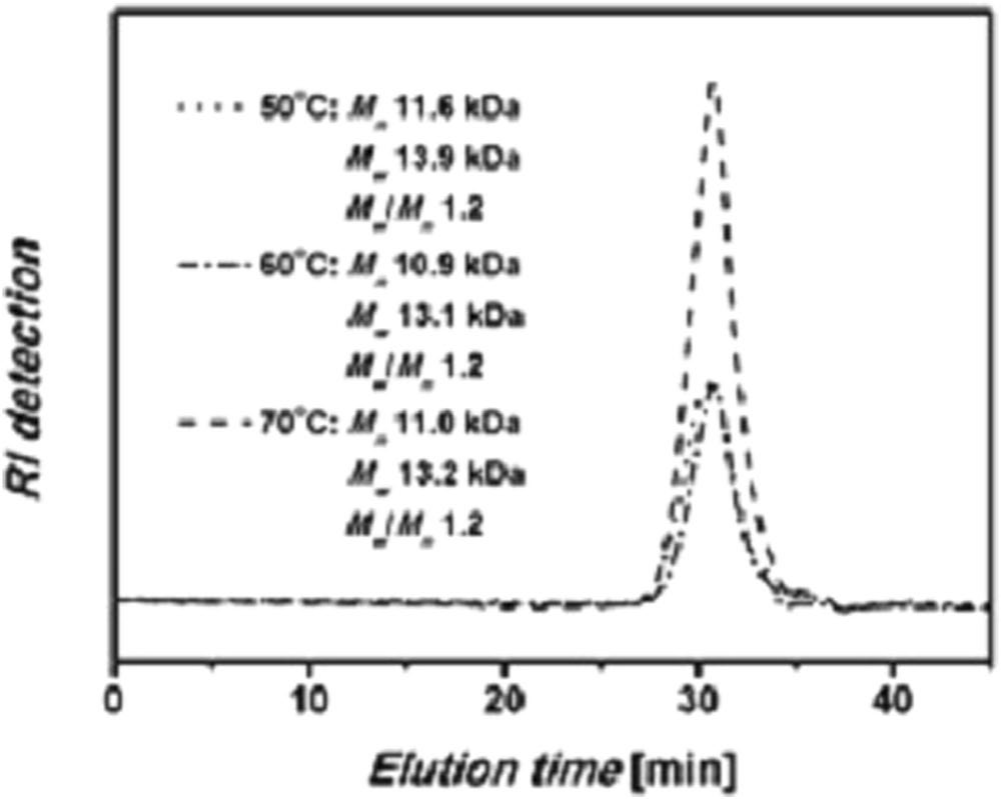
GPC spectra of polymer **PII** at different polymerization temperatures (DMF, on the basis of polystyrene standards).

The results of polymerization at different temperatures were summarized in Table 2. And Figs 5 and 6 show the yields and molecular weight of typical polymer PII at different polymerization temperatures. The yields of **PII** were very low at 30 °C and 40 °C, whereas the polymerizations were significantly accelerated at 50 °C, 60 °C and 70 °C and polypyrazoles with quite high yields were obtained. Particularly, the yield of **PIII** was more than 90%. The yield of **PI** was the lowest at the same polymerization temperature. As shown in Fig. 4, the GPC spectra of typical polymer **PII** obtained at different polymerization temperatures. We could find that the mean molecular weights of **PII** were similar at three polymerization temperatures, and the molecular weight distributions were all about 1.2. However, as shown in Table 2, the mean molecular weights of **PI** were decreased with the increase of polymerization temperatures, indicating that higher temperature went against forming of macromolecular polymer of aliphatic 1,7-octadiyne with diazo compounds. For the polymer **PV**, the mean molecular weights were similar at three polymerization temperatures, but the *M*_*w*_ */M*_*n*_ is about 1.3, which wider than other polymers.

The polymers were soluble in DMF and DMSO, but showed low solubility in the commonly used solvents like THF, acetone, acetonitrile, and chloroform. As expected, the solubility of **PI** was higher than that of other polymers, because of its flexible alkyl chain. In addition, the solubility of **PIII** was higher than **PII**, which is likely due to the flexible alkyl chains of diyne **II**.

**Figure 7:**
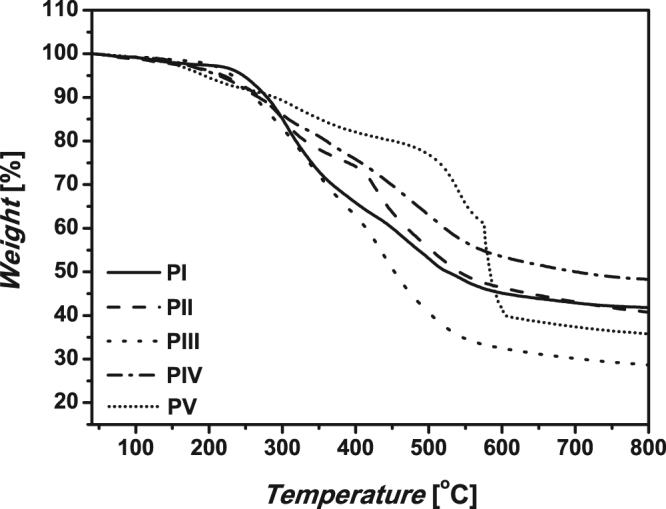
TGA thermograms of **PI** - **PV** recorded under nitrogen at a heating rate of 10 °C/min.

The thermal properties of the polymers were evaluated by DSC (Table 3) and TGA (Table 3, Fig. 7). The glass transition temperatures (*Tg*) of five polymers (**PI–PV**) were more than 120 °C, indicating that the five polymers maintained the glassy state at higher temperature. As shown in Fig. 5, a small weight loss at lower temperature can be attributed to the evaporation of solvents and degradation of small molecular weight residues. Polypyrazoles lost about 10% of their weight at temperatures as high as ~290 °C. The four polymers (**PI–PIV**) showed a high rate of weight loss at about 305 °C, likely caused by the decomposition of the pyrazole moieties. The polymers **PII**, **PIII**, **PIV** showed another high rate of weight loss at about 425–500 °C (Fig. 6, example of **PII**), but not polymer **PI**, indicating it was caused by the decomposition of benzyl groups. The polymer **PV** showed a first high rate of weight loss at about 350 °C, higher than others. And two successive high rate of weight loss happened as high as about 545 °C and 574 °C, much higher than that of the other polymers. It indicates pyrazole moieties directly connected onto benzene ring will greatly improve the thermal stability of the polymer.

**Table 3:** Thermogravimetric Analyses and Crystallization Properties of the polypyrazoles^a^.

Polymer	10% wt loss^b^ (°C)	The first high rate of wt loss^b^ (°C)	The second high rate of wt loss^b^ (°C)	*Tg* ^c^ (°C)
**PI**	279	305	—	151
**PII**	296	302	435	158
**PIII**	280	325	440	120
**PIV**	313	303	500	147
**PV**	290	350	545/574	148

^a^Polymerization conditions:[Diyne]:[Dialdehyde] = 1:1, 48 h, 60 °C, in 25 ml DMF. ^b^The data about the weight loss were measured by TGA and DTG(±1 °C). ^c^Glass transition temperatures (*Tg*) were measured at a heating rate of 20 °C/min using DSC (±1 °C) and collected from the second heating scans.

## Discussion

Previous research demonstrated that terephthalaldehyde was nearly completely converted to the hydrazone with *p*-toluenesulfonylhydrazide and subsequently into the corresponding diazo compound. Therefore, terephthalaldehyde was chosen as the precursor of the diazo compound. Five alkynes from 4 types, as shown in Fig. 2, were brought or synthezied to demonstrate the scope of the one-pot strategy. 1,7-octadiyne is an alkyl acetylene. And diyne **I** and diyne **II** were acetylenes with electron-acceptor substituents but diyne **I** with a rigid structure; diyne **II** with a rigid structure and a flexible alkyl linker. Diyne **III** is AB-type monomers with one terminal aldehyde group and the other alkynyl group. 1,4-diethynylbenzene with totally rigid structure is an acetylene substituted by phenyl group. NMR and IR data suggested that all five of alkynes were transformed to polypyrazoles. It indicates the one-pot strategy has a potentially versatility with tolerant of most functional groups and performs well in mild environment. Successful synthesis of **PIV** suggested the two click reactions involved in this process might be independent and do not affect each other.

Yields of the polypyrazoles varied in the order: **PIII**>**PII**>**PV**>**PIV**>**PI**, which is attribute to reactivity order of the diynes resulting from the abilities of various substituted groups to activate the carbon-carbon multiple bonds^22^. None of conjugation between the triple bond and the substituents displays a low reactivity while Electron-acceptor substituents raise the reactivities of carbon-carbon multiple bonds. However, diyne I and diyne II, having similar structures, shown different reactivities. One possible explanation is that diyne **II** has a flexible group in the middle of the two rigid benzene ring, which may facilitate the 1,3-dipolar cycloaddition reaction. It should be noticed the yields and molecular weights of **PI** were decreased with the increase of polymerization temperatures. we can infer that higher temperature goes against the cycloaddition between the polymers with terminal groups.

The solubility of the polymers is a very important property for their preparation, characterization, processing and application. The test results shown the polymers were soluble in DMF and DMSO, but showed low solubility in the commonly used solvents like THF, acetone, acetonitrile, and chloroform. It can be inferred that H-bonds between pyrazole moieties enhance the intramolecular force of the polymer molecules. We also applied the XRD to the polymers, and no crystallinity was found in the polymers. The DSC and TG results indicates pyrazole moieties directly connected onto benzene ring will greatly improve the thermal stability of the polymer.

## Conclusions

We have used click reactions in a one-pot procedure to synthesize 3,5-disubsituted polypyrazoles with high yields, high molecular weights and narrow molecular weight distribution. The one-pot procedure allowed the *in situ* formation of diazo compounds, which were directly used for the 1,3-dipolar cycloaddition with various alkynes. The developed method could be used to prepare a variety of polypyrazoles with distinct structures.

## Method

### Materials

Terephthalaldehyde (Alfa Aesar, 98%), p-toluenesulfonylhydrazide (Alfa Aesar, 98%), 1,7-octadiyne (Alfa Aesar, 98%), 1,4-diethynylbenzene (Tokyo Chemical Industry, 98%), hydroquinol (Tianjin Guangfu Fine Chemicals Institute, 99%), bisphenol-A (Tianjin Fuchen Chemical Reagent), propargyl bromide (Alfa Aesar, 80% in toluene, stab. with MgO), sodium hydroxide (Beijing Chemical Industry Factory, AR), and calcium chloride anhydrous (Beijing Chemical Industry Factory, AR) were purchased from the respective suppliers. All the solvents used in this study were purchased from Beijing Chemical Industry Factory, including petroleum ether, ethyl acetate, methanol, acetone, dimethyl sulfoxide (DMSO), N,N-dimethylformamide (DMF), tetrahydrofuran (THF), and dichloromethane (DCM). Unless specifically stated, these commercially available reagents and solvents were directly used without further purification.

### Analysis and characterization

Thin layer chromatography (TLC) analysis was performed on silica gel plates and preparative chromatography was carried out on silica gel columns. Molecular weights (Mw and Mn) and molecular weight distributions (Mw/Mn) were measured by gel permeation chromatography (GPC) using a system equipped with a Waters 2414 refractive index detector and an Waters Styragel HT3, HT4, HT5 column, 7.8 × 300 mm^2^, which has a 5 μm bead size with a measurable molecular weight range from 5000 to 40,0000. DMF was used as the eluent at a flow rate of 1 ml/min at 50 °C and a set of monodisperse linear polystyrenes were used as the calibration standards. ^1^H NMR (400 MHz) and ^13^C NMR (100 MHz) were recorded in DMSO-*d6* on a Bruker AV-400 spectrometer, and tetramethylsilane (TMS; δ = 0) was used as the internal standard. IR spectra were obtained on a Thermal Electron Corporation Nicolet 8700/Continuum XL FT-IR spectrophotometer. Differential scanning calorimetry (DSC) was performed with a NETZSCH DSC 204 F1 at a heating rate of 20 °C/min and a flow rate of 50 ml/min under nitrogen atmosphere. Thermal stabilities were evaluated by thermogravimetric analysis (TGA) and differential thermogravimetry analysis (DTG), which were carried out under nitrogen (flow rate at 45 ml/min) on a Perkin Elmer TGA analyzer at a heating rate of 10 °C /min from 25 to 1000 °C.

### One-Pot Synthesis and Characterization of Polypyrazoles

Scheme 1 shows the three steps in this one-pot synthesis and all reactions were carried out in an open atmosphere. Due to the limited sources of diyne starting materials, we have successfully synthesized three types of ether diynes and one alkyne monomer (Scheme 2: diyne **I**, diyne **II**, alkyne **III**) according to reference^20^.

### Synthesis of Polypyrazoles (PI, PII, PIII and PV)

A typical three-step experimental procedure for the synthesis of polypyrazole (**PII**) in one-pot is given below as an example. First, terephthalaldehyde (0.2683 g, 2.000 mmol) and p-toluenesulfonylhydrazide (0.7470 g, 4.011 mmol) were added to DMF (25 ml) in a three-necked round bottomed flask (100 ml). The corresponding hydrazone formation was complete after reaction at 20 °C for 3 h under constant stirring, as indicated by TLC analysis. An aqueous solution of sodium hydroxide (4.5 ml, 4.5 mmol NaOH) was added to the solution subsequently. The mixture was stirred for 30 min at the same temperature, and the solution became reddish. TLC analysis indicated the hydrazone was converted to 1,4-phenyldiazomethane. To obtain the yield of hydrazone formation, the hydrazone was isolated by precipitation with water and its yield was more than 95%.

After the first two steps, diyne I (0.3725 g, 2.001 mmol) was added to the mixture. The temperature was raised to 50 °C, and the reaction was allowed to proceed for 48 h with stirring. The resulting mixture was poured into 400 mL deionized water, and an emulsion was obtained. Calcium chloride anhydrous (4.4325 g, 40 mmol) was added to the emulsion as a demulsifier. The precipitate was dissolved in DMSO, and precipitated by methanol, collected by filtration, and dried at 45 °C for 12 h to constant weight, yielding polypyrazole(**PII**).

### Characterization of Polypyrazoles PI, PII, PIII and PV

**PI** is a saffron powder. When prepared at 50 °C, the yield was 61.02% with *Mw* 16.7 kDa, *Mn* 14.1 kDa and *Mw/Mn* 1.2. When prepared at 60 °C, the yield was 68.65% with *Mw* 14.6 kDa, *Mn* 12.1 kDa and *Mw*/*Mn* 1.2. When prepared at 70 °C, the yield was 59.12% with *Mw* 11.3 kDa, *Mn* 9.0 kDa and *Mw*/*Mn* 1.3. ^1^H NMR (400 MHz, DMSO-*d6*), δ (TMS, ppm): 12.76 (NH, heterocylic), 7.90 (Ar H), 7.47 (CH, heterocylic), 2.41, 2.20, 1.71, 1.52 (-CH2-, aliphatic). ^13^C NMR (100 MHz, DMSO-*d6*), δ (TMS, ppm): 153.09 (-C=N-, heterocylic), 129.83 128.70, 127.50 (Ar C), 100.18 (CH, heterocylic), 71.40, 62.55, 21.12, 17.34 (-CH2-, aliphatic). IR (KBr), v (cm^−1^): 3290 (≡C-H), 3026 (C-H, aromatic), 2933 (-CH2-, str, asym), 2858 (-CH2-, str, sym), 2117 (C≡H), 1621, 1509(C=C).(see also Figure SI-6, Figure SI-7, Figure SI-15 in the Supporting Information).

**PII** is a yellow powder. When prepared at 50 °C, the yield was 77.47% with *Mw* 13.9 kDa, *Mn* 11.6 kDa and *Mw*/*Mn* 1.2. When prepared at 60 °C, the yield was 84.78% with *Mw* 13.1 kDa, *Mn* 10.9 kDa and *Mw*/*Mn* 1.2. When prepared at 70 °C, the yield was 95.01% with *Mw* 13.2 kDa, *Mn* 11.0 kDa and *Mw*/*Mn* 1.2. ^1^H NMR (400 MHz, DMSO-*d6*), δ (TMS, ppm): 13.26, 13.14 (NH, heterocylic), 7.93, 7.83, 7.00 (Ar H), 6.82 (CH, heterocylic), 5.03, 4.72 (OCH2), 3.52 (CCH). ^13^C NMR (100 MHz, DMSO-*d6*), δ (TMS, ppm): 152.48 (-C=N-, heterocylic), 125.18, 115.76, 115.49, 102.55 (Ar C), 93.44 (CH, heterocylic), 78.16, 77.83 (CCH), 55.79 (OCH2). IR (KBr), v (cm^−1^): 3210 (≡C-H), 3139 (C-H, aromatic), 2917 (C-H, str, asym), 2861 (C-H, str, sym), 2119(C≡H), 1620, 1506 (C=C), 1218 (phenyl-O-CH2).

**PIII** is a pale yellow powder. When prepared at 50 °C, the yield was 98.92% with *Mw* 13.2 kDa, *Mn* 11.8 kDa and *Mw*/*Mn* 1.2. When prepared at 60 °C, the yield was 96.77% with *Mw* 14.4 kDa, *Mn* 11.9 kDa and *Mw*/*Mn* 1.2. When prepared at 70 °C: the yield was 98.92% with *Mw* 13.4 kDa, *Mn* 10.9 kDa and *Mw*/*Mn* 1.2. ^1^H NMR (400 MHz, DMSO-*d6*), δ (TMS, ppm): 13.26, 13.12 (NH, heterocylic), 7.82, 7.11, 6.93 (Ar H), 6.82 (CH, heterocylic), 5.05, 4.74 (OCH2), 3.51 (CCH), 1.59 (-CH3). ^13^C NMR (100 MHz, DMSO-*d6*), δ (TMS, ppm): 155.02, (-C=N-, heterocylic), 129.43, 127.37, 125.32, 114.07 (Ar C), 104.46 (CH, heterocylic), 79.15, 78.05 (CCH), 55.05 (OCH2), 30.66 (-CH3). IR (KBr), v (cm^−1^): 3194 (≡C-H), 3031(C-H, aromatic), 2963, 2865 (-CH3), 2120 (C≡H), 1606, 1508 (C=C), 1222 (phenyl-O-CH2).(see also Figure SI-9, Figure SI-10, Figure SI-16 in the Supporting Information).

**PV** is a yellow powder. When prepared at 50 °C, the yield was 79.23% with *Mw*13.0, *Mn* 10.4 kDa and *Mw*/*Mn* 1.3. When prepared at 60 °C, the yield was 84.51% with *Mw* 13.1 kDa, *Mn* 10.4 kDa and *Mw*/*Mn* 1.3. When prepared at 70 °C: the yield was 82.75% with *Mw* 12.9 kDa, *Mn* 10.2 kDa and *Mw*/*Mn* 1.3. ^1^H NMR (400 MHz, DMSO-*d6*), δ (TMS, ppm): 13.45 (NH, heterocylic), 7.96, 7.57 (Ar H), 7.33 (CH, heterocylic), 4.26 (CCH). ^13^C NMR (100 MHz, DMSO-*d6*), δ (TMS, ppm): 150.75, (-C=N-, heterocylic), 125.44, 125.19, (Ar C), 104.00 (CH, heterocylic), 82.55, 76.12 (CCH). IR (KBr), v (cm^−1^): 3283 (≡C-H), 3015 (C-H, aromatic), 2913 (C-H, str, asym), 2857 (C-H, str, sym), 1908 (C≡H), 1607, 1501 (C=C).(see also Figure SI-13, Figure SI-14, Figure SI-18 in the Supporting Information).

### Synthesis of Polypyrazoles (PIV)

The monomer alkyne III possesses not only an alkynyl group but also an aldehyde group, so no other aldehyde was introduced into the procedure. The alkyne III (0.3206 g, 2.001 mmol) and p-toluenesulfonylhydrazide (0.3738 g, 2.007 mmol) were added to DMF (25 mL) in a three-necked round bottomed flask (100 ml). After reacting at 20 °C for 3 h with stirring, an aqueous solution of sodium hydroxide (2.25 ml, 2.25 mmol NaOH) was added. TLC analysis indicated corresponding hydrazone was converted to the diazo compound. The temperature was then raised to 50 °C, and the mixture was stirred for another 48 h. The resulting mixture was poured into 300 mL deionized water, and the resulting emulsion was de-emulsified by anhydrous calcium chloride (3.3245 g, 30 mmol). The precipitates were collected by filtration and dried for 12 h at 45 °C to constant weight to obtain polypyrazole **PIV**.

**PIV** is an off-white powder. When prepared at 50 °C, the yield was 76.02% with *Mw* 10.4 kDa, *Mn* 9.3 kDa and *Mw*/*Mn* 1.1. When prepared at 60 °C, the yield was 73.10% with Mw 11.3 kDa, Mn 9.2 kDa and *Mw*/*Mn* 1.1. When prepared at 70 °C, the yield was 87.71% with *Mw* 10.3 kDa, *Mn* 8.4 kDa and *Mw*/*Mn* 1.1. ^1^H NMR (400 MHz, DMSO-*d6*), δ (TMS, ppm): 13.14, 12.99 (NH, heterocylic), 7.64, 7.10 (Ar C), 6.70 (CH, heterocylic), 5.10, 4.83 (OCH2), 3.55 (CCH). ^13^C NMR (100 MHz, DMSO-*d6*), δ (TMS, ppm): 157.83 (-C=N-, heterocylic), 129.56 127.77, 126.33, 115.14, 114.29 (Ar C), 101.68 (CH, heterocylic), 79.16, 78.69 (CCH), 55.18 (OCH2). IR (KBr), v (cm^−1^): 3278 (≡C-H), 3130(C-H, aromatic), 2921 (C-H, str, asym), 2861 (C-H, str, sym), 2120 (C≡H), 1613, 1510 (C=C), 1244 (phenyl-O-CH2).(see also Figure SI-11, Figure SI-12, Figure SI-17 in the Supporting Information).

## Electronic supplementary material

Supporting Information
