# Supplementary material for: One-Pot Synthesis of Polypyrazoles by Click Reactions
Source: Sci Rep. 2017 Oct 5;7:12712. doi: 10.1038/s41598-017-12727-3 (PMC5629195; doi:10.1038/s41598-017-12727-3)
Supplement: Supplementary file 1 — Supporting Information [file 41598_2017_12727_MOESM1_ESM.pdf]

Supporting Information

One-Pot Synthesis of Polypyrazoles by Using Click Reaction

S. Wang, B. Cheng*

Key Laboratory of Beijing City on Preparation and Processing of Novel Polymer Materials,

Beijing University of Chemical Technology, Beijing 100029, China

***Corresponding to chengb@mail.buct.edu.cn**

Contents:

Synthesis of Alkyne Monomers: P.3- P.4

^{1}H, ^{13}C NMR spectra of alkyne monomers: P.5- P.6

^{1}H, ^{13}C NMR spectra of polymers: P.6- P.9

IR spectra of alkyne monomers and polymers: P.9- P.10

TGA —DTG curves of polymers: P.11-P12

Synthesis of Alkyne Monomers

Three arylpropargyl ethers (diyne **I,** diyne **II,** alkyne **III)** were prepared by simple etherification of different aromatic phenols and propargyl bromide under reflux in acetone (Scheme 1). Detailed procedure for the preparation of diyne **I** was given below as an example. Acetone was dried with K_2CO_3 overnight and distilled. Into 250 mL round-bottom flask were added hydroquinone (2.20g, 20 mmol) and anhydrous potassium carbonate (11.06 g, 80 mmol) in 100 mL of dry acetone. The mixture was stirred at ambient temperature for 0.5 h. Then propargyl bromide (6.69 g, 45mmol) was added dropwise within 0.5 h under stirring. Afterwards, the mixture was refluxed for an additional 24 h. The mixture was cooled to room temperature and filtered, and the filtrate was concentrated on a rotary evaporator. The residue was dissolved in 100 mL DCM and washed with 100 mL of water three times and 100 mL of saturated brine once. The organic phase was dried over $MgSO_4$. After filtration and evaporation, the crude product was purified on a silica gel column using petroleum ether/ethyl acetate (6:1 by volume) as the eluent. In this procedure, anhydrous potassium carbonate and propargyl bromide were used in excess. A white solid was obtained in 85% yield. ^{1}H NMR (400 MHz, DMSO-d_6), δ (TMS, ppm): 6.93 (s, 1H, Ar H), 4.73(d, 1H, J =2.3 Hz, OCH_2), 3.53 (t, 1H, J =2.3 Hz, CCH). ^{13}C NMR (100 MHz, DMSO-d_6), δ (TMS, ppm): 151.64, 115.78 (ArC), 79.61, 78.15 (CCH), 55.80(OCH_2). IR (KBr), v (cm^{-1}): 3275(≡C—H), 2130(C≡C), 1507, 1450 (O-CH_2), 1378, 1283, 1222, 827.

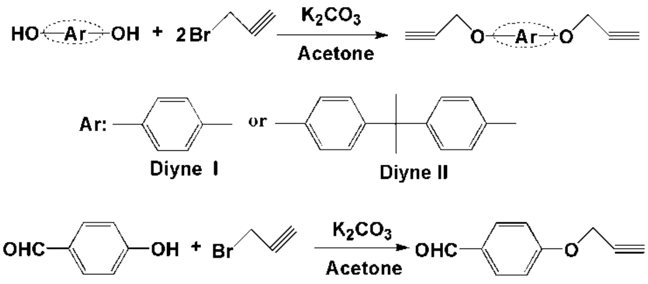

SCHEME 1 Synthesis of monomer diyne **I,** diyne **II** and alkyne **III**

As Scheme 1 shows, monomer diyne **II** was prepared from the reaction of bisphenol A with propargyl bromide by the same synthetic procedures. A white solid

was obtained in 89% yield. ^{1}H NMR (400MHz, DMSO-d_6), δ (TMS, ppm): 7.12 (d, *J*=8.8 Hz, 1H, Ar H), 6.88(d, *J*=8.8 Hz, 1H, H Ar-O), 4.74(d, 1H, *J*=2.3 Hz, OCH_2), 3.53(t, 1H, *J*=2.3 Hz, CCH), 1.59 (s, 1H, CH_3). ^{13}C NMR (100 MHz, DMSO-d_6), δ (TMS, ppm): 155.15, 143.12, 127.51, 114.29(Ar C), 79.63, 78.20 (CCH), 55.18 (OCH_2), 41.14 (CCH_3), 30.81 (CH_3). IR (KBr), *v* (cm^{-1}): 3284 (≡C—H), 3036, 2971 (-CH_3), 2874, 2119 (C≡C), 1605, 1507, 1451 (O-CH_2), 1385, 1367, 1288, 1264, 1217, 1182, 1020, 833.

Monomer alkyne **III** was prepared from parahydroxybenzaldehyde and propargyl bromide by the similar synthetic procedures. The only difference between alkyne **III** and diyne **I,** diyne **II** is that alkyne **III** contain both an aldehyde group and an alkynyl group, so the amount of anhydrous potassium carbonate and propargyl bromide in half, anhydrous potassium carbonate (6.22g, 45mmol), propargyl bromide (3.72g, 25mmol). A white solid was obtained in 80% yield. ^{1}H NMR (400 MHz, DMSO-d_6), δ (TMS, ppm): 9.90(s, 1H, CHO), 7.89(m, 2H, Ar H), 7.17(d, 2H, *J*=8.7 Hz, Ar H), 4.95(d, 2H, *J* =2.4 Hz, OCH_2), 3.53(t, 1H, *J*=2.3 Hz, CCH). ^{13}C NMR (100 MHz, DMSO-d_6), δ (TMS, ppm): 191.49 (CHO), 162.16, 131.80, 130.29, 115.44 (Ar C), 78.98, 78.67(CCH), 55.79 (OCH_2). IR (KBr), *v* (cm^{-1}): 3212 (≡C—H), 3078, 2928, 2832, 2122 (C≡C), 1682 (CHO), 1603, 1577, 1506, 1426 (O-CH_2), 1380, 1252, 1170, 1022, 829.

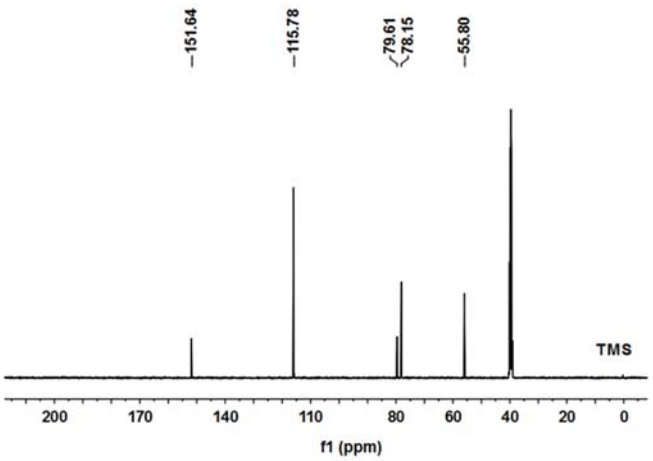

Figure SI-1 ^{13}C NMR spectrum (100 MHz, DMSO-d_6) of monomer diyne **I**

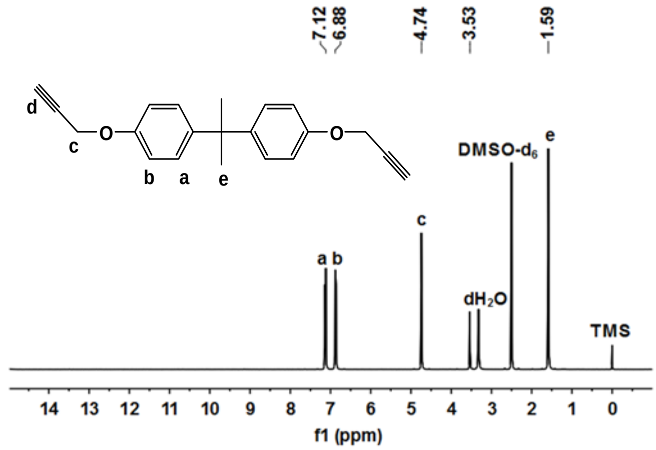

Figure SI-2 ^{1}H NMR spectrum (400 MHz, DMSO-d6) of monomer diyne **II**

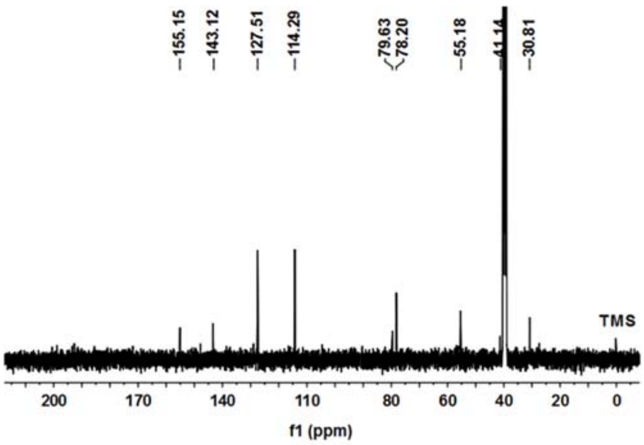

Figure SI-3 ^{13}C NMR spectrum (100 MHz, DMSO-d6) of monomer diyne **II**

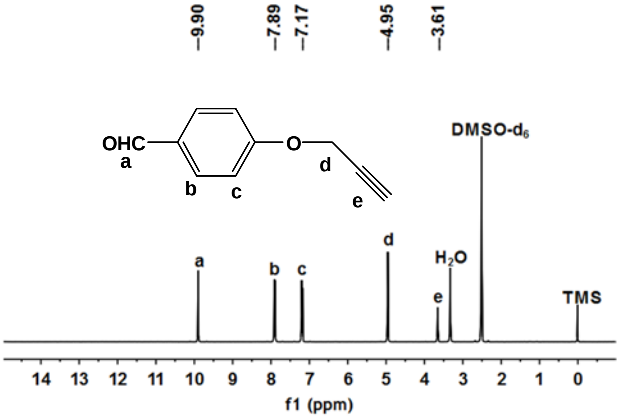

Figure SI-4 ^{1}H NMR spectrum (400 MHz, DMSO-d6) of monomer alkyne **III**

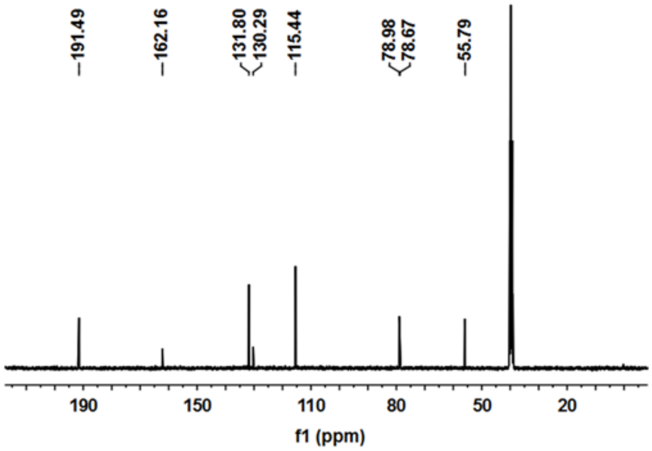

Figure SI-5 ^{13}C NMR spectrum (100 MHz, DMSO-d$_6$) of monomer alkyne **III**

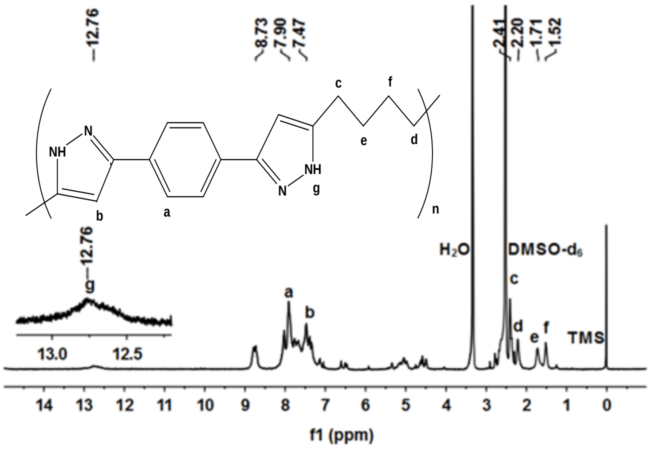

Figure SI-6 ^{1}H NMR spectrum (400 MHz, DMSO-d$_6$) of polymer **PI**

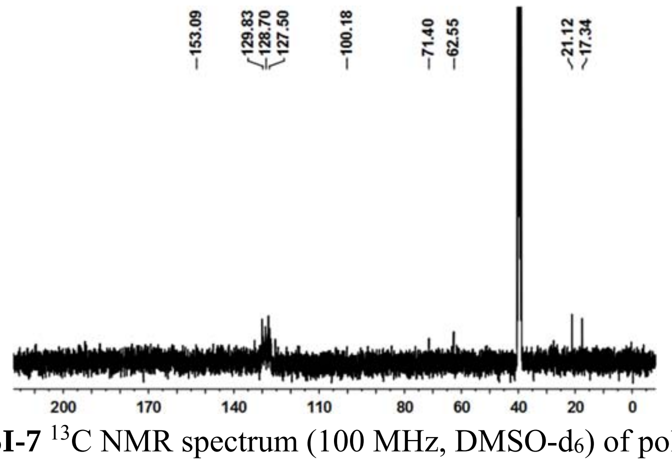

Figure SI-7 ^{13}C NMR spectrum (100 MHz, DMSO-d$_6$) of polymer **PI**

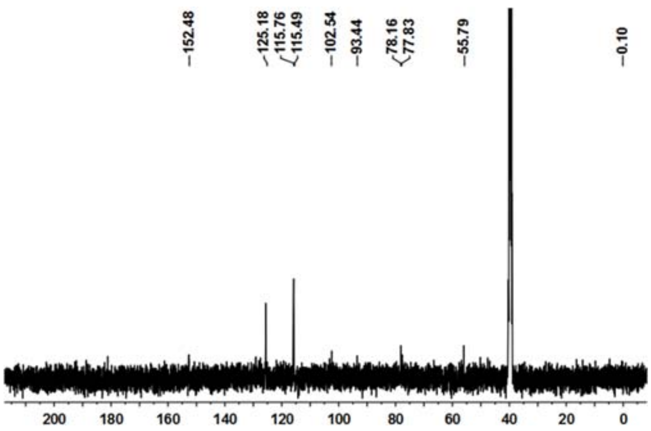

Figure SI-8 ^{13}C NMR spectrum (100 MHz, DMSO-d$_6$) of polymer **PII**

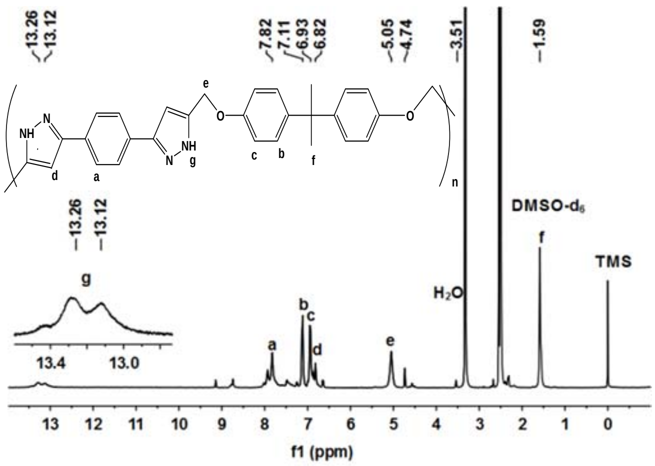

Figure SI-9 ^{1}H NMR spectrum (400 MHz, DMSO-d$_6$) of polymer **PIII**

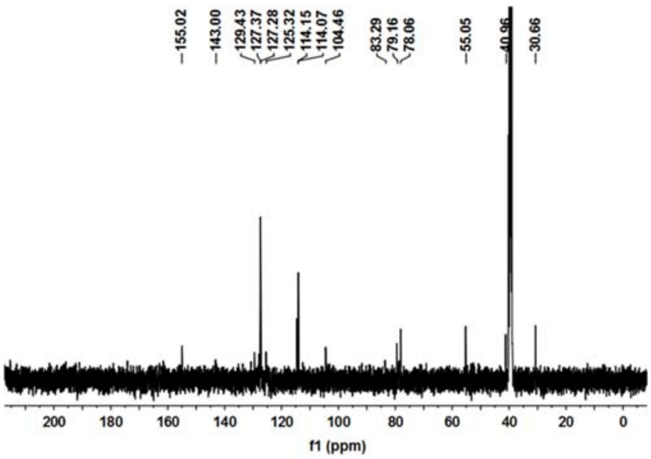

Figure SI-10 ^{13}C NMR spectrum (100 MHz, DMSO-d$_6$) of polymer **PIII**

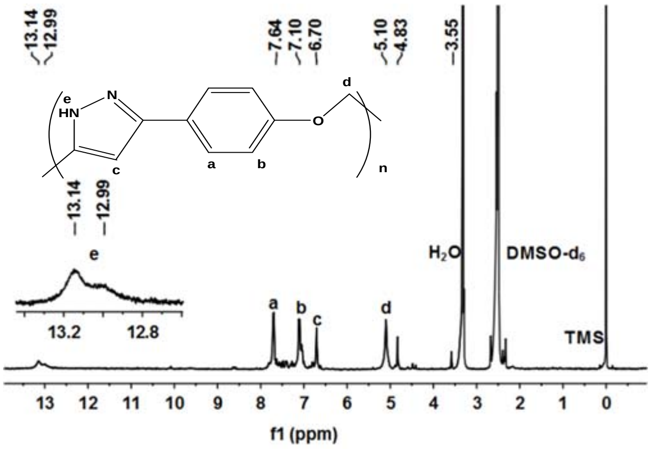

Figure SI-11 ^{1}H NMR spectrum (400 MHz, DMSO-d$_6$) of polymer **PIV**

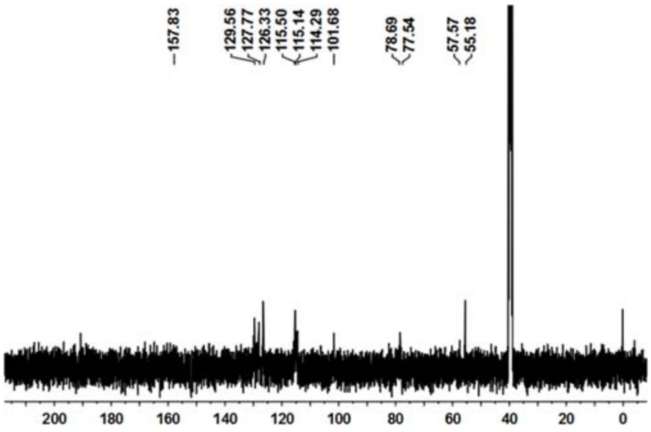

Figure SI-12 ^{13}C NMR spectrum (100 MHz, DMSO-d$_6$) of polymer **PIV**

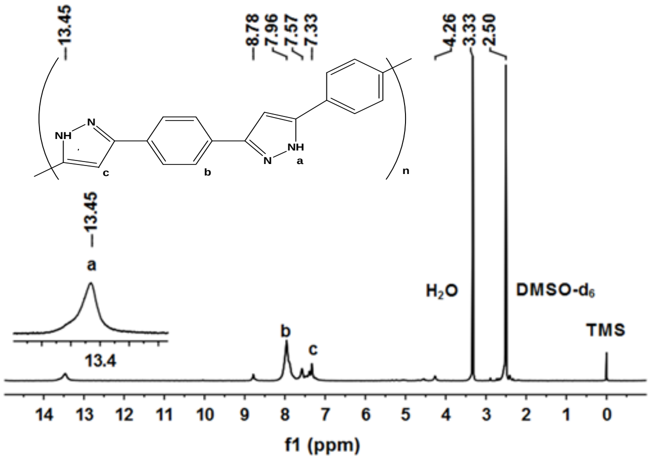

Figure SI-13 ^{1}H NMR spectrum (400 MHz, DMSO-d$_6$) of polymer **PV**

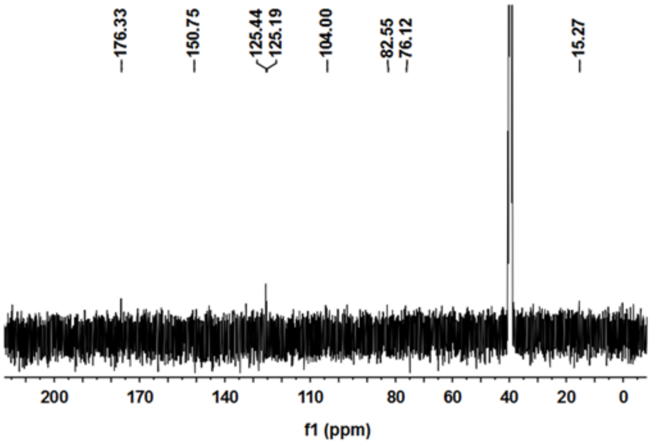

Figure SI-14 ^{13}C NMR spectrum (100 MHz, DMSO-d6) of polymer **PV**

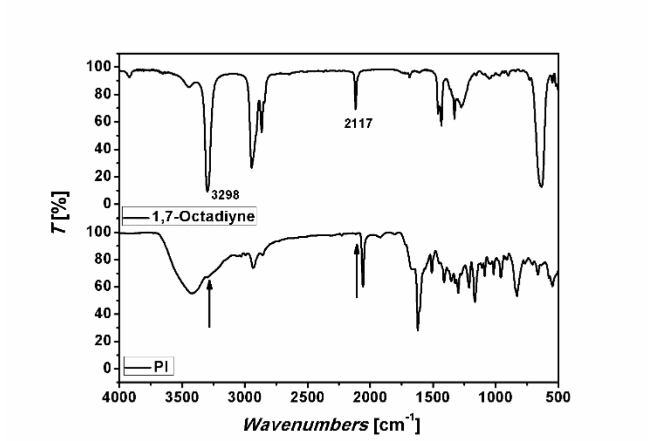

Figure SI-15 IR spectra (KBr) of 1,7-octadiyne **[**3298 cm^{-1} (≡C—H), 2117 cm^{-1} (C≡C)]and its polymer **PI**

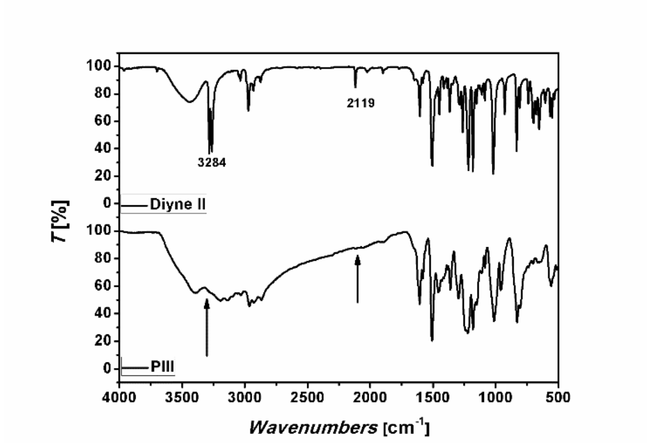

Figure SI-16 IR spectra (KBr) of diyne **II [**3284 cm^{-1} (≡C—H), 2119 cm^{-1} (C≡C)] and its polymer **PIII**

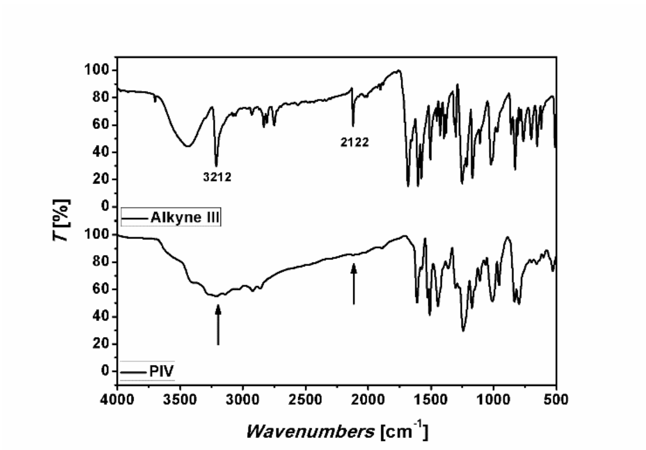

Figure SI-17 IR spectra (KBr) of alkyne **III** [3212 cm^{-1} (≡C—H), 2122 cm^{-1} (C≡C)] and its polymer **PIV**

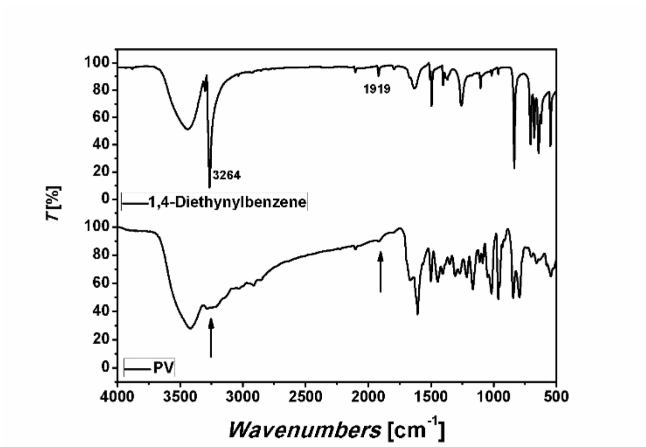

Figure SI-18 IR spectra (KBr) of 1,4-Diethynnylbenzene [3264 cm^{-1} (≡C—H), 1919 cm^{-1} (C≡C)] and its polymer **PV**.

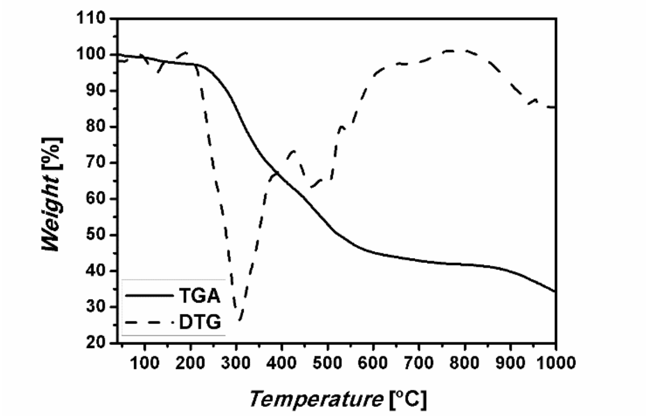

Figure SI-19 TGA thermograms of **PI** and its DTG curve recorded under nitrogen at a heating rate of 10 °C /min.

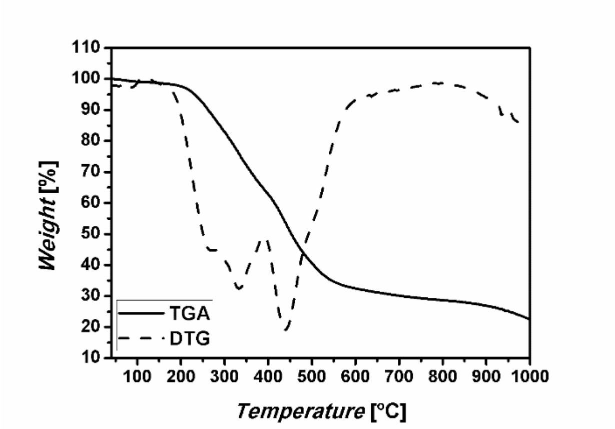

Figure SI-20 TGA thermograms of **PIII** and its DTG curve recorded under nitrogen at a heating rate of 10°C /min.

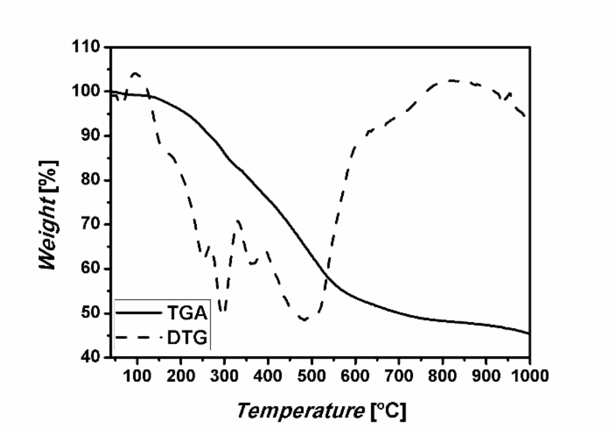

Figure SI-21 TGA thermograms of **PIV** and its DTG curve recorded under nitrogen at a heating rate of 10°C /min.

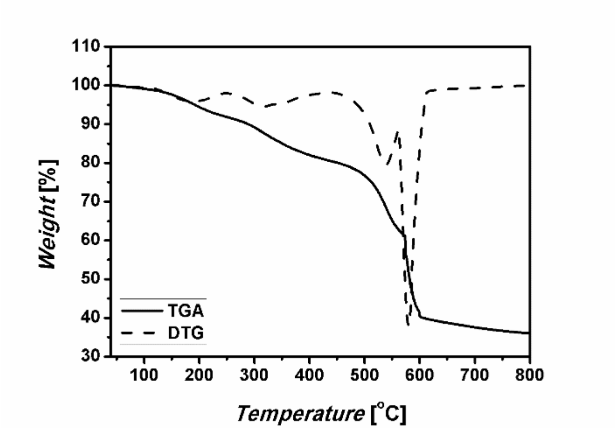

Figure SI-22 TGA thermograms of **PV** and its DTG curve recorded under nitrogen at a heating rate of 10°C /min.
